# The Essential Strategies to Mitigate Cardiotoxicity Caused by Doxorubicin

**DOI:** 10.3390/life13112148

**Published:** 2023-10-31

**Authors:** Aleksey Michailovich Chaulin

**Affiliations:** 1 Department of Histology and Embryology, Samara State Medical University, Samara 443099, Russia; alekseymichailovich22976@gmail.com; 2 Department of Clinical Chemistry, Samara State Medical University, Samara 443099, Russia

**Keywords:** cardiotoxicity, doxorubicin, risk factors, cardiovascular risk, cardiovascular diseases, cardioprotection strategies

## Abstract

The study of mechanisms underlying cardiotoxicity of doxorubicin and the development of strategies to mitigate doxorubicin-induced cardiotoxicity are the most relevant issues of modern cardio-oncology. This is due to the high prevalence of cancer in the population and the need for frequent use of highly effective chemotherapeutic agents, in particular anthracyclines, for optimal management of cancer patients. However, while being a potent agent to counteract cancer, doxorubicin also affects the cardiovascular systems of patients undergoing chemotherapy in a significant and unfavorable fashion. Consecutively reviewed in this article are risk factors and mechanisms of doxorubicin cardiotoxicity, and the essential strategies to mitigate cardiotoxic effects of doxorubicin treatment in cancer patients are discussed.

## 1. Introduction

Cardiovascular diseases (CVDs) and cancer not only are the leading causes of mortality and disability but also are closely related, and this relationship is bidirectional [1,2,3]. A range of risk factors, such as smoking, obesity, lack of physical activity, as well as family history (genetic predisposition), are common for the development of CVDs and cancer [1,2,3,4,5]. The interaction of CVDs and cancer is an illustrative example of iatrogenic comorbidity, i.e., drugs that are highly effective in treating one disease increase the risk of the other [6,7].

Improvements in chemotherapy and treatment regimens (protocols) have contributed to a gradual increase in survival rates in cancer patients: 16.9 million in 2019 vs. presumably 22.1 million by 2030 [8]. However, cancer patients are at high risk of cardiovascular death since CVDs are one of the main and dangerous complications of anticancer drugs. A large retrospective cohort study including 36,232 adult cancer survivors showed that patients with cardiovascular risk factors (≥2) had an almost twofold increased risk of coronary heart disease, stroke, and cardiomyopathy/heart failure compared with controls (odds ratio 1.83–2.59, *p* < 0.01). The 8-year overall survival rate in cancer patients with CVDs was 60% compared with 81% in patients without CVDs (*p* < 0.01) [9]. Therefore, the development and search for cardioprotective strategies is a priority for modern health care. 

Anthracyclines, among which doxorubicin is the best known, are the main components of polychemotherapy protocols, despite the fact of their long-time use [10]. Anthracyclines exhibit antitumor properties through four main mechanisms: (1) nucleic acid (DNA and RNA) production disruption through intercalation between base pairs, (2) topoisomerase II inhibition (which leads to DNA breaks and prevents its reparation by ligation), (3) cause modification of histone proteins, thereby inhibiting DNA repair, and (4) increased iron-mediated formation of free radicals (reactive oxygen species (ROS)), which leads to damage to nucleic acid and intracellular protein–lipid structures [10,11]. The above mechanisms are also closely related and potentiate each other, thereby locking “vicious circles” in pathogenesis. For example, topoisomerase inhibition leads to ROS accumulation, which causes DNA strands to break and trigger apoptotic pathways, leading to cell death. Anthracyclines are commonly used in chemotherapy regimens for leukemia, lymphoma, sarcoma, and breast cancer. According to various data, 5–15% of patients treated with anthracyclines develop chronic heart failure (CHF) [12]. Different pathogenetic mechanisms underlie cardiotoxic effects of different classes of anticancer agents, which determine variability of clinical and laboratory signs, risk stratification, and treatment [13]. Clinical and laboratory presentation of doxorubicin-induced cardiotoxicity is not limited to CHF; it also includes asymptomatic left ventricular (LV) systolic dysfunction, atrial and ventricular arrhythmia, acute myocarditis and pericarditis, and myocardial infarction (MI) [11,13,14,15,16]. Understanding the key pathophysiological mechanisms underlying anthracycline cardiotoxicity is an important step towards the development of cardioprotective pathogenetic therapy [10]. 

Major risk factors for anthracycline cardiotoxicity include type and cumulative dose of chemotherapy, regimen and route of administration, combination with other cardiotoxic drugs or with radiation therapy on the chest area, female sex, hypertension, coronary artery disease (CAD), obesity, type 2 diabetes mellitus (T2DM), CHF, prior therapy with anthracyclines or radiation therapy, valve diseases, baseline LV function, African-American descent, age >65 years or <18 years, renal failure, concomitant use of other anticancer drugs (cyclophosphan, methotrexate, trastuzumab, and others), electrolyte imbalance (hypokalemia and hypomagnesemia) and, likely, genetic and environmental risk factors [11,13,17]. The recommended optimal strategy of anthracycline pre-treatment patient management is complete elimination (or at least mitigation) of the negative impact of major cardiovascular risk factors, i.e., hypertension, previous hemodynamically significant structural heart disease (heart defects, angina, arrhythmias, and CHF), metabolic disorders (diabetes and obesity), as well as lifestyle modification, including smoking cessation, body weight normalization, and optimization of physical activity. Risk stratification using biomarkers or echocardiographic data can be useful in identifying patients who can benefit from more aggressive chemotherapy (or for whom it should be limited) and the maximum use made of cardioprotective strategies and more intensive patient surveillance during cancer treatment [13,18,19]. In accordance with the current guidelines of cardio-oncology group of the Heart Failure Association of the European Society of Cardiology (2020) [13], the risk stratification of anthracycline cardiotoxicity should be performed according to the following proforma (Table 1). 

**Figure 1:**
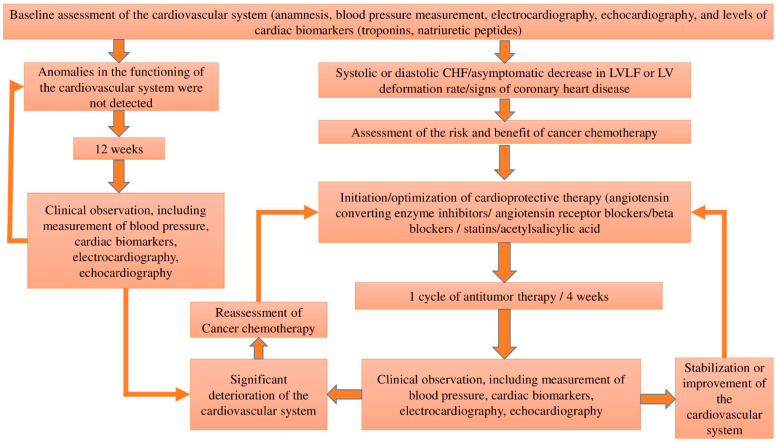
CVD monitoring during anti-cancer chemotherapy with potentially cardiotoxic drugs, adapted from [20].

According to the expert opinion, the percentage probability of developing potential cardiotoxicity for each risk group is as follows: low risk (<2%), medium risk (2–9%), high risk (10–19%), and very high risk (≥20%) [13]. The likelihood of developing potential cardiotoxicity should be taken into account in patient management. Thus, low-risk patients are recommended to continue chemotherapy and have their CVDs monitored according to local, national, and international guidelines [11,13]. Intermediate-risk patients require closer monitoring of their CVDs during treatment, or their referral to specialist cardiology or cardio-oncology units should be considered. High- and very-high-risk patients should be referred to a specialist cardio-oncology unit, where an individualized follow-up plan for the period of chemotherapy is developed [13,19]. The following CVD monitoring scheme during chemotherapy is proposed (Figure 1). 

The main strategies to prevent and/or minimize the cardiotoxic effects of anthracyclines are considered to be as follows: (1) early diagnosis, (2) elimination of common risk factors, (3) reduction of cumulative doxorubicin dose, (4) administration of liposomal doxorubicin, (5) exosomal delivery of doxorubicin, (6) use of dexrazoxane, and (7) pathogenetic and symptomatic therapy with a range of therapeutic agents, including statins and basic CHF therapy drugs (beta-blockers, renin–angiotensin–aldosterone system (RAAS) inhibitors, and specifically, mineralocorticoid receptor antagonists, as well as myocardial cytoprotectors, etc.). Currently, the dominant management strategy for cardio-oncology patients is their risk stratification by cardiotoxicity severity with subsequent specific monitoring and treatment [13]. Nevertheless, as evidenced in practice, the current cardioprotective measures are not sufficient, which determines the need for further research on new targets and drugs. Therefore, in addition to the current opportunities, this article will discuss certain promising cardioprotection fields and new possible targets. 

## 2. Early Diagnosis of Anthracycline Drugs Cardiotoxicity

Cardiac imaging plays an important role in the diagnosis of cardiotoxicity. Echocardiography to assess left ventricular ejection fraction (LVEF) should be performed before initiating anthracyclines and repeated at regular intervals depending on the patient’s cardiotoxicity risk. Thus, in low-risk cases or in the absence of abnormal changes in cardiovascular system function during anthracycline chemotherapy, functional tests can be performed every 12 weeks, whereas in high-risk patients or established cardiovascular system abnormalities during chemotherapy, a 4-week interval between tests is required (Figure 1) [21,22]. 

Echocardiography with 3D LVEF assessment, contrast echocardiography with analysis of global longitudinal strain (GLS) of the left ventricle have higher sensitivity and are useful in detecting asymptomatic and subclinical LV dysfunction [22,23]. GLS assessment, previously only available in expert centers, is now present in ultrasound systems of all leading manufacturers and has become widely available [23,24]. Patients with multiple cardiovascular risk factors, low baseline GLS (or decrease by >12% from the baseline during treatment), or low LVEF at baseline (<53%) may benefit from more frequent echocardiography testing during anthracycline treatment [21,22,23].

According to a recent statement by the Heart Failure Association (HFA), the European Association for Cardiovascular Imaging (EACVI) and the Cardio-Oncology Council of the European Society of Cardiology (ESC), echocardiographic monitoring should be performed during the first year (after 6 months with a high baseline risk of cardiotoxicity or after 12 months with a low or medium baseline risk of cardiotoxicity) after completion of chemotherapy [25], because during this period, signs of doxorubicin-induced cardiotoxicity are very often manifested (myocardial remodeling with a decrease in left ventricular mass and cardiomyocyte atrophy, LV dysfunction with a decrease in the LVEF) [26,27]. In addition, with long-term follow-up after the completion of antitumor therapy (for several years), repeated control echocardiography is recommended in certain populations, such as young patients who received high cumulative doses of anthracyclines (>400 mg/m^2^ of doxorubicin), patients with significant pre-existing CVD, female cancer survivors planning to become pregnant or at the end of the first trimester of pregnancy, and individuals who plan to compete in high-intensity exercises, for example, in marathons, cycling, triathlon, etc. [18,25,28]. 

An important role in the early diagnosis and monitoring of doxorubicin-induced cardiotoxicity is played by laboratory diagnostics based on the determination of serum levels of specific markers of myocardial damage and dysfunction. According to recent clinical recommendations of the Cardio-Oncology Study Group of the Heart Failure Association and the Cardio-Oncology Council of the European Society of Cardiology, cardiac troponins and natriuretic peptides are the most preferred biomarkers for the management of patients with doxorubicin-induced cardiotoxicity [29]. It is recommended to use the following algorithm (Table 2) for laboratory diagnostics of doxorubicin-induced cardiotoxicity.

The development of anthracycline-induced LV dysfunction is also evidenced by increased levels of moderately sensitive cardiac troponins (MSCTs), highly sensitive cardiac troponins (HSCT), and natriuretic peptides, which can be used for early diagnosis [30,31,32,33,34]. According to recent data, HSCTs are superior to MSCTs in assessing the cardiotoxic effects of doxorubicin since these can reveal subclinical cardiomyocyte damage at the stages of cardiotoxic action when MSCTs are still negative [30,31,32,33]. Integrated use of functional and laboratory diagnostic testing is most effective. Thus, simultaneous assessment of GLS combined with determination of HSCT I level had 93% sensitivity and 91% negative predictive value in predicting further LV dysfunction [33]. Thus, cardiomarkers, including cardiac troponins and natriuretic peptides, are the most promising clinical tool for both baseline risk assessment and markers of early damage or deformation of the heart, which may predict subsequent changes in LVEF and development of HF in various cardiotoxic cancer treatments, including doxorubicin therapy [29]. 

In addition to natriuretic peptides, a number of researchers suggest using myeloperoxidase, high-sensitivity C-reactive protein, soluble fms-like tyrosine kinase-1 (sFlt-1), placental growth factor (PGF), growth differentiation factor-15 (GDF-15), galectin-3, arginine–nitric oxide metabolites, heart-type of fatty acid binding protein (h-FABP), glycogen phosphorylase BB, topoisomerase 2ß, immunoglobulin E (IgE), microribonucleic acids (microRNAs), and tumor growth factors; however, so far, the diagnostic significance of these markers has not been sufficiently proven and requires further clarification [29,35,36,37,38,39,40]. 

## 3. Eliminating the Risk Factors Common for CVDs and Cancer 

Elimination of the risk factors common for CVDs and cancer, in particular, smoking cessation, normalization of body weight, and metabolic disorders, as well physical activity optimization, is one of the priority therapeutic and prevention strategies [1,2,41]. Lack of physical activity is closely related to the other risk factors, including excessive weight and metabolic disorders (diabetes mellitus, metabolic syndrome, etc.) that can arise in cancer patients and contribute to the additional cardiotoxicity risk. Cancer patients decrease their physical activity by 2 h/week prior to and after the diagnosis, which is 11% less than their baseline capabilities (*p* < 0.05) [42]. Moreover, it was found that female patients with breast cancer gain 2.7 kg (range 2.5–6.2 kg) during chemotherapy [43].

In the general population, the cardioprotective effect of aerobic training is well studied; however, the specific mechanism of cardioprotection in cancer patients is not yet fully understood. There is an opinion that physical exercise reduces anthracycline cardiotoxicity by reducing the drug concentration in cardiomyocytes, decreasing apoptotic signaling and ROS generation, which leads to improvement of endothelial function [44] and increasing anthracycline washout from cells [41]. Increased production of antioxidants in cardiomyocyte mitochondria observed during physical activity results in a cardioprotective effect against anthracycline-mediated ROS level increase [44]. However, data on benefits of physical activity are ambiguous since intense and/or prolonged physical training causes cardiomyocyte damage in healthy athletes, with a tenfold or more increase in HSCT level [45,46,47]. On this basis, it can be assumed that even moderate physical activity, taking into account additional myocardial weakening, can have a detrimental effect to cardiomyocytes, and the development of a physical exercise plan during chemotherapy should be individually based. 

The American College of Sports Medicine (ACSM) has published a consensus on the safety of exercise for specific groups of cancer patients and cancer survivors, confirming overall safety and effectiveness of physical training [48]; however, experts also note that further clinical research is needed to determine the optimal exercise strategy.

## 4. Reduction of Cumulative Dose and Prolonged Intravenous Infusion of Anthracyclines

The maximum cumulative dose of anthracyclines is clearly associated with higher rates of CHF morbidity and mortality. Cumulative doxorubicin doses of 550 mg/m^2^ or more frequently cause doxorubicin-induced cardiomyopathy (and heart failure), so these should be limited to 450 mg/m^2^. At the same time, it is emphasized that there is no completely safe doxorubicin dose. In addition, doxorubicin cardiotoxicity is determined by the rate of drug administration. Thus, a recent large Cochrane meta-analysis established that continuous long-term infusions of the drug for at least 6 h (6 to 96 h) are less likely to lead to the development of LV dysfunction as compared with shorter-term drug infusions (odds ratio 0.29, 95% confidence interval 0.09–0.81). Notably, continuous infusion of doxorubicin for 48–96 h was associated with a decrease in myocardial morphological changes without differences in anti-tumor efficacy [49]. Since the severity of cardiotoxic effects of anthracyclines depends on their peak concentration, and their antitumor efficacy is associated with the average plasma concentration (area under the pharmacokinetic curve), fractional or prolonged administration appears to be a simple measure to reduce the risk of LV dysfunction and CHF without loss of antitumor efficacy. A study of different schemes of anthracycline administration where endomyocardial biopsy was used showed that administration of 20 mg/m^2^ once weekly resulted in a significant myocardial damage reduction compared with a single infusion of 60 mg/m^2^ once every 3 weeks [49]. 

## 5. Liposomal Doxorubicin

Liposomes are the most common and well-studied nanocarriers for targeted drug delivery. They stabilize therapeutic compounds by overcoming barriers to cellular and tissue drug uptake and improving the biodistribution of compounds to target sites in vivo. The unique ability of liposomes to capture both lipophilic and hydrophilic compounds makes it possible to encapsulate in these vesicles drugs with various physical and chemical properties [50]. Pegylated liposomal doxorubicin allows more efficient drug delivery, reducing its cardiotoxicity [50,51,52]. Nevertheless, due to the lack of studies on different aspects of liposomal doxorubicin cardiotoxicity, there is a need for well-powered studies in patient populations. 

## 6. Exosomal Delivery of Doxorubicin

Exosomal delivery of doxorubicin is a very promising therapeutic approach to reduce the risk of side effects from ongoing anticancer chemotherapy. Exosomes are extracellular natural vesicles of microscopic size (30–200 nm), used as alternative means of delivering a number of molecules, in particular, chemotherapeutic drugs to tumor cells. Exosomes consist of a membrane that is formed as a result of the insertion into the endosomal membrane and a cavity that contains host cell molecules: proteins, lipids, RNA, etc. Exosomes have the ability to cross histo-hematic barriers, in particular the blood–brain and hemato-thymus barriers and can be internalized into various cells of the human body through endocytosis, pinocytosis, and phagocytosis [53,54,55,56]. Relatively recently, it was found that endosomes can be manipulated, in particular, to change their composition (add various low-molecular therapeutic agents) and route. A number of recent studies have demonstrated the high efficacy of exosomal doxorubicin for the treatment of breast and ovarian cancer [55], malignant glioma [56], osteosarcoma [57], and colorectal cancer [58]. Exosomal delivery of doxorubicin avoids doxorubicin-induced cardiotoxicity, which is due to a decrease in the penetration of the drug through the endothelial cells of myocardial vessels. So, according to G. Toffoli et al., the accumulation of doxorubicin is reduced by 40% with the exosomal method of delivery of this drug, and this does not lead to a decrease in the therapeutic efficacy of doxorubicin against tumor cells [59,60]. Thus, liposomal doxorubicin is safer than free doxorubicin and can be considered as a very promising therapeutic approach aimed at minimizing cardiotoxic effects.

## 7. Dexrazoxane

Dexrazoxane is an ethylenediaminetetraacetate (EDTA) derivative reducing ROS level in myocardium by chelating iron to reduced iron complexes with anthracyclines [61]. Dexrazoxane is a clinically proven agent for the prevention of doxorubicin-induced cardiotoxicity. The U.S. Food and Drug Administration (FDA) guidelines currently recommend dexrazoxane for breast cancer patients with high cumulative anthracycline doses, as well as for children when the anthracycline dose exceeds 300 mg/m^2^ [62]. There is a concern that dexrazoxane will limit antitumor efficacy and increase myelosuppression, the incidence of infectious complications, and the development of secondary malignancies [63]. Dexrazoxane exhibits cardioprotective effects not only in anthracycline-induced cardiotoxicity but also in a number of other CVDs. L. Zhou examined the cardioprotective effects of dexrazoxane and the underlying mechanisms of cardioprotection in a rat model of MI. The authors demonstrated that dexrazoxane has a cardioprotective effect in MI, and a proposed mechanism was associated with apoptosis inhibition and increased neovascularization [63].

The protective mechanisms of dexrazoxane are multifaceted and not fully understood. The study evaluated how dexrazoxane affects activation and transmission of intracellular kinase signalling, in particular, protein kinases B (Akt) and extracellular signal-mediated kinases (Erk 1 and Erk 2), the key functions of which are to regulate cell survival and proliferation as well as tolerance to ischemia. The protective properties of dexrazoxane were found to be related to the activation of Akt, Erk 1, and Erk 2. In addition, the results of this study demonstrated that the cardioprotective effect of dexrazoxane persists for a long period [64].

## 8. Therapeutic Options for Anthracycline-Induced Cardiotoxicity

### Statins

Statins are thought to exhibit pleiotropic effects, i.e., in addition to their main lipid-lowering action, they additionally have anti-inflammatory, antioxidant, and other properties. In addition, they can play a role in preventing cardiotoxicity of anthracyclines. Statins can reduce oxidative stress and inflammatory response, being a part of the pathogenesis of many CVDs. Statins can also improve endothelial function and nitric oxide metabolism [65]. A small observational study of breast cancer patients who received anthracyclines combined with atorvastatin throughout and after treatment demonstrated a lower risk of developing CHF [66]. In patients receiving high doses of atorvastatin (40–80 mg), LVEF increased despite the presence of a large number of cardiovascular risk factors [67].

Experimental studies in pigs and rodents demonstrated that bolus atorvastatin administration during ischemia limits cardiac cell death and reduces MI area by enhancing activity of AMP-mediated protein kinase (AMPK), the primary function of which is to regulate cellular energy balance [68]. 

L. Zhang et al. indicated that atorvastatin protects cardiomyocytes from oxidative stress by inhibiting LOX-1 expression and apoptosis [69]. Pre-treatment of cardiomyocytes with fluvastatin can mitigate doxorubicin-induced cardiotoxicity through significant reduction of oxidative stress and inhibition of inflammatory and apoptotic pathways, as evidenced by reduced activity of cardiac lipid peroxidation, expression of nitrotyrosine, tumor necrosis factor-alpha (TNF-a), and apoptosis marker Bax [70].

At the same time, some evidence suggests that statins exhibit pro-oxidant rather than antioxidant properties. Thus, by inhibiting HMG-CoA reductase, these agents inhibit coenzyme Q (ubiquinone) synthesis, which is an important antioxidant and energy compound for muscle tissues, including myocardium [71]. According to other data, patients receiving statins have higher levels of HSCT than those not treated with statins, which may indicate cardiomyocyte damage [72,73,74,75]. In another study, ventricular cardiomyocytes of newborn laboratory mice were treated with atorvastatin and pravastatin for 48 h. Atorvastatin, but not pravastatin, inhibited intracellular cardiac Akt/mTOR kinase pathway signaling and disrupted mitochondrial ultrastructure in cardiac myocytes, which, according to the authors, may have important clinical implications [75]. 

## 9. Beta-Adrenoblockers

Beta-adrenoblockers are an important part of drug therapy of CHF of various geneses. Carvedilol is a third-generation non-selective beta-adrenoblocker that reduces ROS level, prevents mitochondrial dysfunction, and inhibits lipid peroxidation, contributing to preserved systolic and diastolic myocardial function during antracycline treatment [76]. In the OVERCOME study, patients with hematologic malignancies who received carvedilol and lisinopril had higher LVEF and lower rate of CHF events and deaths as compared with those not treated with these drugs [76]. However, in the CECCY trial of women receiving anthracyclines, carvedilol failed to prevent a decrease in LVEF, although its administration was associated with a lower incidence of diastolic dysfunction and decrease in blood MSCT levels compared with the control group [77].

Nebivolol is another third-generation beta-adrenoblocker with vasodilator and antioxidant properties. Similar to carvedilol, nebivolol may also have cardioprotective effects against anthracycline-induced cardiotoxicity. In a small double-blind study of patients receiving anthracyclines, nebivololol prevented a decrease in LVEF more significantly than in the placebo group and also reduced cardiac dysfunction markers levels—natriuretic peptides (NT-pro-BNP) after 6 months [78].

Other beta-adrenoblockers that do not have vasodilator or antioxidant properties do not exhibit cardioprotective activity [79]. 

## 10. RAAS Inhibitors

ACE inhibitors (ACEi) and angiotensin II receptor blockers (ARBs) are neurohormonal blocking agents used to prevent cardiac remodeling [80]. The efficacy of ACEi and BRAs has also been studied as a treatment of cancer patients with cancer-induced HCF. ACE inhibitors protect myocardium from free radicals produced by anthracyclines, exhibiting antifibrotic effect and normalizing calcium homeostasis in cardiomyocytes exposed to anthracyclines [81]. In a clinical trial, cancer patients receiving lisinopril (n = 114) had stable LVEF and a lower risk of adverse CV events compared with the placebo group [81]. 

In the OVERCOME study, it was demonstrated that pretreatment with carvedilol combined with lisinopril was effective in preventing the decrease in LVEF [76]. The PRADA study showed that in breast cancer patients receiving candesartan during anthracycline chemotherapy, the LVEF decrease was less severe, whereas metoprolol did not exhibit the same effect. However, it was noted that metoprolol combined with candesartan produced no additional benefits for cardiovascular system status and function [79].

## 11. The Use of Human-Induced Pluripotent Stem Cells (hPSCs)-Cardiomyocytes 

Recently human-induced pluripotent stem cells (hPSCs-cardiomyocytes) have been widely used as experimental (preclinical) models to study the pathophysiological mechanisms of doxorubicin-induced cardiotoxicity and to search for new effective targets for therapeutic effects [82,83,84]. Thus, thanks to the use of hPSCs-cardiomyocytes, the mechanisms of doxorubicin-induced cardiotoxicity have been well studied: oxidative stress, mitochondrial dysfunction, disruption of calcium homeostasis, as well as changes in the expression levels of genes and proteins that trigger apoptotic death of cardiomyocytes [83,84,85]. In addition, hPSCs-cardiomyocytes were used to study some cardioprotective strategies: reduction of cardiotoxicity due to modification of doxorubicin (analogues), targeted delivery of anthracycline chemotherapy drugs specifically into tumor cells, and the introduction of cardioprotective agents in combination with doxorubicin [83]. 

## 12. The Role of Genome-Wide Association Studies (GWAS Studies) in Identifying the Risk of Doxorubicin-Induced Cardiotoxicity

Advances in pharmacogenomics have been driven by both candidate genes and genome-wide associations (GWAS) and have resulted in the identification of numerous single-nucleotide polymorphisms (SNPs) statistically related to efficacy and toxicity of drugs, including doxorubicin [86]. This is of important practical importance, as it will help identify patients at higher risk of developing cardiotoxicity. Recently, E. Christidi et al. established that the missense variant of the retinoic acid receptor gene-γ (RARG) (S427L; rs2229774) increases susceptibility to cardiotoxicity induced by doxorubicin. The missense variant of the RARG gene (S427L; rs2229774) was associated with higher oxidative stress and cardiomyocyte apoptosis compared with the control group (wild-type animal cardiomyocytes). Genetic correction of RARG-S427L to the wild type led to a decrease in doxorubicin-induced double-stranded DNA breaks, production of reactive oxygen species, and cell death [86]. T. Magdy et al. studied the mechanism by which SNP (rs2229774) in RARG enhances susceptibility to doxorubicin-induced cardiotoxicity: inhibition of topoisomerase 2ß (TOP2B) expression and activation of the cardioprotective extracellular regulated kinase (ERK) pathway [87]. Thus, clinical genetic screening of rs2229774 before doxorubicin chemotherapy may be useful to minimize the risk of cardiotoxic effects.

## 13. New Potential Targets and Drugs to Prevent Cardiotoxicity

Given the lack of efficacy of existing cardioprotective drugs, there is a need for new, more effective drugs to decrease cardiotoxicity of anticancer drugs. In a single article, it is hardly possible to discuss the variety of currently proposed targets and drugs to minimize cardiotoxicity. As an example, this article briefly discusses some of the most promising targets/drugs for reducing doxorubicin cardiotoxic effects: thrombopoietin, sestrins, ghrelin, sirtuins, natural phytocompounds (resveratrol, flavonoids, Vitamin E, and lotusine), and mitochondrial-targeted cardioprotective strategies.

***Thrombopoietin.*** Thrombopoietin (TPO) is an endogenous cytokine involved in hematopoiesis (a megakaryocyte/platelet formation stimulator), exhibits anti-apoptotic activity, and stimulates angiogenesis. In several experimental studies, cardioprotective effects of TPO have been detected. K. Li et al. were the first to present evidence that TPO is a protective agent against doxorubicin-induced cardiotoxicity. Cardioprotective effects of TPO were studied on the cell line of neonatal myocytes H9C2 (in vitro) and an experimental model (mouse model of DOX-induced acute cardiomyopathy (in vivo)). The use of TPO reduced apoptosis of cardiac myocytes (decreased expression of annexin V, active caspase-3, and normalization of mitochondrial membrane potential), improved myocardial morphology (reduced cytoplasmic vacuolization, myofibrillar loss, and apoptosis), and functional parameters of the myocardium (increased heart rate, fractional shortening, and cardiac output) in laboratory animals [88]. 

K. Chan with colleagues demonstrated that TPO prevents cardiac cells from acute and chronic damage caused by doxorubicin. A possible molecular mechanism of TPO action was activation of kinase signaling pathways Akt and Erk-1,2 that limited cardiomyocyte apoptosis. In addition, there was an improvement in the morphology and function of the myocardium in laboratory animals treated with TPO [89]. In another study, K. Chan et al. also noted similar beneficial effects of TPO in a rat model of myocardial infarction [88]. On the basis of the results obtained, the investigators concluded that TPO can be used as a component of doxorubicin-induced CHF therapy [88,89,90].

A recent study conducted by H. Wang et al. confirmed the presence of the previously described cardioprotective effects of thrombopoietin in doxorubicin-induced cardiotoxicity. Researchers have established that TPO has a protective role in H9C2 cells from autophagy caused by doxorubicin, as well as from apoptosis, and have shown that TPO can act as a cardioprotective drug in doxorubicin-treated patients [91]. Thus, TPO can be considered as one of the promising cardioprotective agents in doxorubicin-induced cardiotoxicity. 

**Figure 2:**
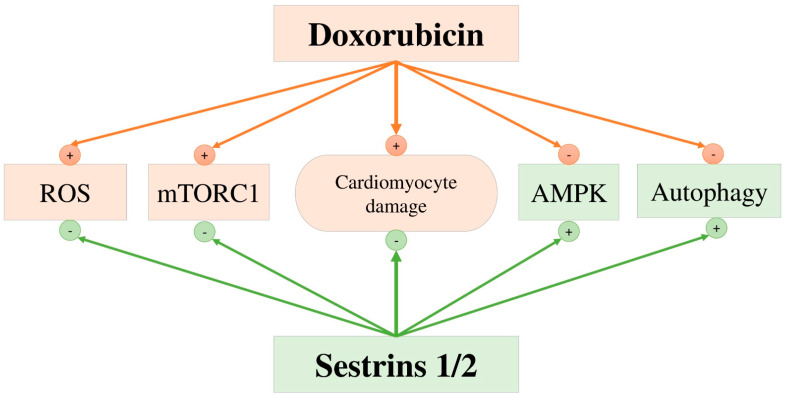
Mechanisms of cardioprotective effects of sestrins.

***Sestrins.*** Sestrin 1 and sestrin 2 (proteins of the sestrin family) are important cardioprotective proteins, coordinating metabolic signaling pathways and autophagy and minimizing cardiac damage caused by doxorubicin cardiotoxicity. Mice with knockout sestrin 1 and sestrin 2 genes were more vulnerable to doxorubicin-induced cardiac diseases, demonstrated more significant cardiac dysfunction, and their histological examination revealed that a greater number of cardiomyocytes were in apoptosis [92,93,94]. Treatment with doxorubicin was accompanied by a decrease in the expression of sestrin 1 and sestrin 2, which was associated with a number of adverse effects (increased doxorubicin-induced oxidative stress, cardiomyocyte apoptosis, cardiac mitophagy, and mitochondrial dysfunction) in laboratory animals [94,95,96,97,98,99]. In a number of studies, it was noted that activation of sestrin 1 prevents oxidative damage and reduces cardiomyocyte apoptosis in response to doxorubicin, which indicates the cardioprotective role of sestrin 1 against doxorubicin-induced toxicity [95,96]. The cardioprotective effects and mechanisms of sestrin 2 have recently been studied by several independent research groups. In general, these researchers concluded that doxorubicin-induced inhibition of sestrin 2 is one of the key factors that enhance apoptosis and oxidative stress of cardiomyocytes [94,97,98,99]. In contrast, increased expression of sestrin 2 has beneficial effects on the myocardium by significantly reducing doxorubicin-induced apoptosis and oxidative stress of cardiac myocytes (Figure 2). Thus, pharmacological interventions aimed at increasing the expression of sestrin 1 and sestrin 2 can be used for the prevention and treatment of doxorubicin-induced cardiotoxicity. 

### 13.1. mTORC1, Mammalian Target of Rapamycin Complex 1; ROS, Reactive Oxygen Species; and AMPK, AMP-Mediated Protein Kinase

***Ghrelin.*** Ghrelin is a multifunctional peptide hormone regulating metabolism and energy homeostasis, playing an important role in cardiovascular system protection [98,99,100]. According to the study of X. Wang, ghrelin inhibited doxorubicin-induced autophagy and reduced cardiomyocyte apoptosis and oxidative stress by increasing the expression and activity of antioxidant defense enzymes [101]. As a result of studying the intracellular signaling pathways of the cardioprotective action of ghrelin, researchers found that this peptide inhibits the formation of ROS and the activity of the enzyme AMPK and stimulates p38-MAPK activity [101]. Z. Xu et al. found that ghrelin increases the activity of antioxidant enzymes (superoxide dismutase and catalase) and increases the expression of mitochondrial antiapoptotic proteins in cardiomyocytes. These molecular effects of ghrelin lead to a decrease in oxidative stress and apoptosis of myocardial cells [102].

M. Elhadidy et al. studied other molecular mechanisms underlying the cardioprotective action of ghrelin. The researchers found that ghrelin increases the expression of vascular endothelial growth factor beta (VEGF-B) and connexin-43 (Cx43) in the myocardium of animals suffering from doxorubicin-induced cardiomyopathy. Increased expression of VEGF-B and Cx43 was associated with normalization of clinical and functional parameters—normalization of electrocardiographic parameters (shortening of the PR, QT, QTC, and ST segment intervals and increased amplitude and decreased duration of the QRS complex) and blood pressure (increase in systolic blood pressure)—and myocardial morphology (reduction of fibrosis and hypertrophy) [103].

Several recent studies by two independent research groups A. Shati et al. and M. Kihara et al. demonstrated pronounced anti-apoptotic and antifibrotic effects of the acylated form of ghrelin [104,105,106]. Thus, recent studies indicate that ghrelin and its acylated form are promising cardioprotective drugs for the management of patients with signs of doxorubicin-induced cardiotoxicity.

***Sirtuins.*** The sirtuin proteins (SIRT1, SIRT3, and SIRT6) that delay premature aging of the cardiovascular system have been one of the promising targets of recent studies, and therefore, are considered as promising targets for therapeutic action. SIRT1, a member of the NAD-dependent sirtuin family of enzymes, plays a key role in aging, metabolism, and myocardial cell survival, and has been shown to prevent cardiac damage [107]. The role of SIRT1 was studied in an experimental study—doxorubicin-induced cardiomyocyte damage in vivo and in vitro. SIRT1 overexpression reduced doxorubicin-induced cardiomyocyte apoptosis and attenuated ROS production. In contrast, the SIRT1 antagonist, niacinamide, increased ROS formation, thereby suppressing the protective effect of SIRT1 in the cultured cardiomyocytes of newborn rats. These results confirm the role of SIRT1 as an important regulator of cardiomyocyte apoptosis in doxorubicin-induced cardiac damage, which may represent a potential therapeutic target for treatment of doxorubicin-induced cardiomyopathy [108]. 

### 13.2. Natural Phytocompounds: Resveratrol, Flavonoids, Vitamin E, and Lotusine

Natural phytocompounds include a very significant amount of chemicals that have traditionally been used by people as food and medicines for the prevention and treatment of a number of diseases, including cardiovascular diseases. Resveratrol (stilben), flavonoids, vitamin E, and lotusine are the main natural phytocompounds with pronounced cardioprotective effects against doxorubicin-induced cardiotoxicity [109,110,111,112,113,114,115,116,117,118,119,120,121,122,123].

***Resveratrol***. Resveratrol, a natural polyphenol found in nuts, fruits, and red wine, plays an important role in preventing cardiovascular disease by reducing oxidative stress. Resveratrol exhibits a protective effect against doxorubicin-induced cardiotoxicity, but its mechanisms are not yet fully understood [109]. Resveratrol is a SIRT1 protein agonist. Pre-treatment of cardiomyocytes with resveratrol reduced the possibility of their death when exposed to doxorubicin. However, protective effects of resveratrol were attenuated with SIRT1 protein inhibition [109]. In another study, Y. Lou demonstrated that resveratrol prevents induced cardiotoxicity due to stress inhibition of endoplasmic reticulum [110]. According to C. Zhang, resveratrol in mice mitigates doxorubicin-induced apoptosis of cardiomyocytes through the SIRT1-mediated p53 deacetylation—a transcription factor, cell cycle regulator [111]. Thus, resveratrol has antioxidant and anti-apoptotic effects, which play an important role in mitigating doxorubicin-induced cardiotoxicity.

**Figure 3:**
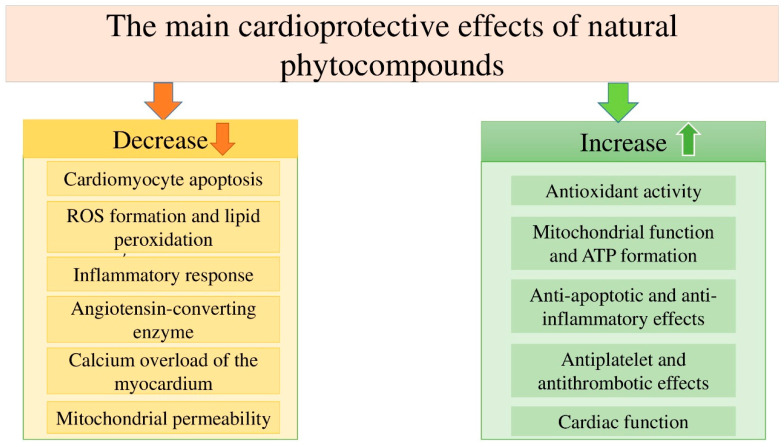
The main effects and mechanisms of cardioprotective action of natural phytocompounds (flavonoids).

***Flavonoids***. Flavonoids are an important group of natural phytocompounds commonly found in many plants. The high pharmacological activity of flavonoids has been documented in many experimental studies (on animal models and cell cultures) and clinical trials on humans [112,113,114,115]. The key pharmacological effects of flavonoids that are important for the prevention and treatment of cardiovascular diseases are inhibition of ROS formation, antiplatelet and antithrombotic activity, inhibition of angiotensin converting enzyme, anti-calcium overload of the myocardium, etc. [115,116,117,118,119]. According to the chemical structure (depending on the C ring and B ring, as well as the unsaturation and oxidation of the C ring), flavonoids are divided into several subclasses: flavonols (quercetin, rutin, myristin, morin, and kaempferol), flavones (apigenin and luteolin), anthocyanins (cyanidin and malvidin), flavanones (hesperetin, naringin, and naringenin), and isoflavonoids (genistin and genistein) [116]. According to the conducted studies, the key effects of flavonoids to combat doxorubicin-induced cardiotoxicity are: (1) a decrease in ROS and lipid peroxidation, (2) an increase in the activity of antioxidant enzymes, (3) a decrease in the inflammatory response in the myocardium, (4) a decrease in the permeability of mitochondria, (5) inhibition of apoptosis of myocardial cells, etc. (Figure 3) [116]. Thus, flavonoids are very useful herbal compounds for the management of patients with doxorubicin-induced cardiotoxicity.

***Vitamin E*.** Vitamin E is a group of natural compounds that are derivatives of tokol. Tocopherols and tocotrienols are the most important biologically active compounds belonging to the group of vitamins E [120,121]. A. Puri et al. were among the first to demonstrate the cardioprotective properties of vitamin E against doxorubicin-induced cardiotoxicity. Thus, treatment of rats with vitamin E led to a decrease in the levels of cardiomarkers (creatine kinase-MV and lactate dehydrogenase), which were increased due to myocardial damage caused by doxorubicin. Along with biochemical parameters of myocardial condition, normalization of electrocardiographic parameters (PR, QT, and ST segments) was noted in those rats that received vitamin E [121]. The main mechanisms underlying the cardioprotective action of vitamin E against doxorubicin-induced cardiotoxicity are: reduction of oxidative stress (neutralization of free radicals and increased catalase activity) and anti-inflammatory effects (decreased expression of inflammatory chemokines MCP-1 and ICAM-1) [122].

***Lotusine.*** Lotusine is also considered one of the promising natural phytocompounds to combat doxorubicin-induced cardiotoxicity. Lotusine is an alkaloid derived from Nelumbo Nucifera (Gaertn.). R. Harishkumar et al. studied the effects and mechanisms of action of lotusine on rat embryonic cardiomyocytes (H9C2). In cells pretreated with lotusine, an increase in the amount of endogenous antioxidants was observed with a decrease in lipid peroxidation, whereas in cells exposed to doxorubicin, the content of antioxidants decreased along with an increase in lipid peroxidation. It was also found that lotusine inhibits cardiomyocyte apoptosis by suppressing the pro-apoptotic Bax gene and caspase-3 [123].

## 14. Mitochondrial-Targeted Cardioprotective Strategies

Among all known cellular organelles and structures of cardiomyocytes, mitochondria are most affected by doxorubicin, and this is accompanied by mitochondrial dysfunction, impaired bioenergetics, depolarization of the mitochondrial membrane potential, and increased ROS production [124]. Therefore, the development of therapeutic drugs aimed at improving mitochondrial functions can be considered as promising cardioprotective strategies. Researchers identify several groups of drugs aimed at improving mitochondrial functions: (1) small molecules (DMX-5804, liensinin, dexmedetomidine, berberine, nicotinamide riboside, and phenylala-nine-butyramide), (2) antioxidants (vitamins C, E, and N-acetylcysteine), (3) microribonucleic acids (microRNAs) (miR-146a, miR-29b, miR-378, and miR-140-5p), and (4) mitochondrial transplantation [124]. A detailed description of the mechanisms of action of these drugs is presented in a recent review [124] devoted to mitochondrial-directed cardioprotective strategies.

## 15. Conclusions

Currently, the main measures to reduce cardiotoxicity of doxorubicin include the following: early diagnosis, reduction of the lifetime cumulative dose, prolongation of intravenous infusions of anthracyclines, elimination of major CVD risk factors (smoking cessation, correction of metabolic disorders, and physical activity), use of dexrazoxane, as well as pathogenetic and symptomatic therapy (beta-adrenoblockers, statins, and RAAS inhibitors). In managing cardio-oncology patients, risk stratification should be performed, with optimal strategies for monitoring and treatment of doxorubicin-associated CVD to be selected. Promising drugs and targets for improving cardioprotective measures include: thrombopoietin, sestrins, ghrelin, sirtuins, natural phytocompounds (resveratrol, flavonoids, vitamin E, and lotusine), and mitochondrial-targeted cardioprotective strategies). Additional well-powered clinical studies are needed to evaluate the role of these compounds.

## Figures and Tables

**Table 1:** Proforma for cardiovascular risk stratification in patients receiving anthracycline chemotherapy. From [13].

Risk Factor	Score
Previous cardiovascular disease:	
- Heart failure or cardiomyopathy	Very high
- Severe heart valve disease	High
- Myocardial infarction or prior coronary revascularization	High
- Stable angina pectoris	High
- Baseline left ventricular ejection fraction < 50%	High
- Borderline left ventricular ejection fraction (50–54%)	Moderate
Cardiac biomarker levels (if available):	
- Increased concentration of cardiac troponins	Moderate
- Elevated levels of natriuretic peptides	Moderate
Demographic and cardiovascular risk factors:	
- Age > 80	High
- Age 65–79	Moderate
- Hypertension	Moderate
- Diabetes mellitus	Moderate
- Chronic kidney disease	Moderate
Previous cancer chemotherapy and radiotherapy:	
- Previous treatment with anthracyclines	High
- Previous radiotherapy to the breast area	High
- Previous chemotherapy with non-anthracyclines	Moderate
Lifestyle-related risk factors:	
- Smoking (current or long-term history)	Moderate
- Obesity (body mass index > 30)	Moderate

Table note: Low risk is the absence of specified risk factors or the presence of one risk factor with an average score. Medium risk is the presence of two to four risk factors with an average score. High risk is the presence of five or more risk factors with an average score or one risk factor with a high score. Very high risk is the presence of one risk factor with a very high score.

**Table 2:** Algorithm for the diagnosis of doxorubicin-induced cardiotoxicity (during and after anthracycline therapy) [29].

Baseline Cardiovascular Risk Assessment (in Accordance with [13])	Cardiomarkers	During Anthracycline Chemotherapy	Following Anthracycline Chemotherapy
Low risk	- Natriuretic peptides (BNP/NT-proBNP) - Cardiac troponins	- Baseline - Before 5th cycle during treatment (optional)	- 12 months after final cycle
Medium risk	- Natriuretic peptides (BNP/NT-proBNP) - Cardiac troponins	- Baseline - Before 5th cycle - Before every cycle (optional)	- 12 months after final cycle
High risk	- Natriuretic peptides (BNP/NT-proBNP) - Cardiac troponins	- Baseline - Before cycles 2, 4, and 6 - Before every cycle (optional)	- 3 and/or 6 months after final cycle - 12 months after final cycle

## Data Availability

Not applicable.
